# Neuromodulation of chronic headaches: position statement from the European Headache Federation

**DOI:** 10.1186/1129-2377-14-86

**Published:** 2013-10-21

**Authors:** Paolo Martelletti, Rigmor H Jensen, Andrea Antal, Roberto Arcioni, Filippo Brighina, Marina de Tommaso, Angelo Franzini, Denys Fontaine, Max Heiland, Tim P Jürgens, Massimo Leone, Delphine Magis, Koen Paemeleire, Stefano Palmisani, Walter Paulus, Arne May

**Affiliations:** 1 Department of Clinical and Molecular Medicine, Sapienza University of Rome and Regional Referral Headache Centre, Sant’Andrea Hospital, Rome, Italy; 2 European Headache Federation, Danish Headache Center, Department of Neurology, Glostrup Hospital, University of Copenhagen, DK-2600 Glostrup, Denmark; 3 Department of Clinical Neurophysiology, Georg-August University, Göttingen, Germany; 4 Department of Medical and Surgical Science and Translational Medicine, Sapienza University of Rome and Pain Therapy Unit, Sant’Andrea Hospital, Rome, Italy; 5 Department of Experimental Biomedicine and Clinical Neurosciences (BioNeC), University of Palermo, Palermo, Italy; 6 Department of Neuroscience and Sensory System, Policlinico General Hospital, Aldo Moro University, Bari, Italy; 7 Department of Neurosurgery, Neurological Institute C. Besta, Milan, Italy; 8 Department of Neurosurgery, Centre Hospitalier Universitaire de Nice, Nice, France; 9 Department of Oral and Maxillofacial Surgery, University Medical Center Hamburg-Eppendorf, Hamburg, Germany; 10 Department of Systems Neuroscience, University Medical Centre Hamburg-Eppendorf, Hamburg, Germany; 11 Department of Neurology, Headache Centre and Pain Neuromodulation Unit, Fondazione Istituto Nazionale Neurologico Carlo Besta, Milano, Italy; 12 Department of Neurology, Headache Research Unit, University of Liège, Liège, Belgium; 13 Department of Neurology, Ghent University Hospital, Ghent, Belgium; 14 Pain Management and Neuromodulation Centre, Guy’s & St Thomas NHS Trust, London, UK

**Keywords:** Chronic headache, Medically intractable headache, Neurostimulation, SPG, DBS, GON, tDCS, TMS, ONS, TENS, VNS, Migraine, Cluster headache, European headache federation

## Abstract

The medical treatment of patients with chronic primary headache syndromes (chronic migraine, chronic tension-type headache, chronic cluster headache, hemicrania continua) is challenging as serious side effects frequently complicate the course of medical treatment and some patients may be even medically intractable. When a definitive lack of responsiveness to conservative treatments is ascertained and medication overuse headache is excluded, neuromodulation options can be considered in selected cases.

Here, the various invasive and non-invasive approaches, such as hypothalamic deep brain stimulation, occipital nerve stimulation, stimulation of sphenopalatine ganglion, cervical spinal cord stimulation, vagus nerve stimulation, transcranial direct current stimulation, repetitive transcranial magnetic stimulation, and transcutaneous electrical nerve stimulation are extensively published although proper RCT-based evidence is limited. The European Headache Federation herewith provides a consensus statement on the clinical use of neuromodulation in headache, based on theoretical background, clinical data, and side effect of each method. This international consensus further gives recommendations for future studies on these new approaches.

In spite of a growing field of stimulation devices in headaches treatment, further controlled studies to validate, strengthen and disseminate the use of neurostimulation are clearly warranted. Consequently, until these data are available any neurostimulation device should only be used in patients with medically intractable syndromes from tertiary headache centers either as part of a valid study or have shown to be effective in such controlled studies with an acceptable side effect profile.

## Background

Although headache is a common disease, its more severe manifestations such as intractable migraine and trigeminal autonomic cephalalgias have a debilitating effect on patients resulting in chronic pain and severe functional impairment. The recent Global Burden of Disease Study 2010 (GBD2010), conducted by the World Health Organization, Lifting the Burden and their partners, estimates a worldwide prevalence of migraine of 14.7%, ranking it third place among the most common diseases and at the seventh place among specific causes of disability and top of all neurological disorders as cause of total years lived with disability [1,2].

Although excellent international guidelines for organisation of headache service and management have been introduced [3-5] there is no single standard of care for patients presenting with primary chronic headache symptoms. For example, treatment choices for acute migraine are based on headache severity, attack frequency, associated symptoms, and co-morbidities. Despite significant improvement in management of migraine, achieving a satisfactory treatment outcome is still a challenge because of inadequate response of medications and difficulty in predicting individual response to a specific agent or dose.

The medical treatment of patients with chronic primary headache syndromes (such as chronic migraine, chronic cluster headache, chronic tension-type headache or hemicrania continua) is particularly challenging as valid studies are few and in many cases even higher doses of preventative medication is ineffective and adverse side effects frequently complicate the course of medical treatment.

Chronic headaches that do not or no longer respond to prophylaxis are commonly encountered at tertiary level headache centres [6]. The vast majority of these patients suffer from medication overuse headache which can and should be alleviated by detoxification, but a subset remains as refractory chronic migraine (RCM) [6]. Although much work has been accomplished, the definition of RCM is still a continuous work in progress [7,8]. Cluster headache as such can also be hard to treat but it may become impossible in chronic cluster headache (CCH) sufferers [9]. Some patients may be intractable to the therapies recommended by national guidelines, and following the need of clinicians the word “intractable” has been defined by Goadsby et al. entitled “Towards a Definition of Intractable Headache for Use in Clinical Practice and Trials” [10]. In these patients, i.e. when the intolerance or lack of responsiveness to conservative treatments is ascertained, surgical options are considered. The options has previously ranged from application of glycerol or local anaesthetics into the cisterna trigeminalis of the Gasserian ganglion; radiofrequency rhizotomy of the Gasserian ganglion or of the trigeminal nerve; microvascular decompression; resection or blockade of the N. petrosus superficialis or of the ganglion sphenopalatinum and to a whole range of other ablative or destructive methods. Case reports of the complete inefficacy of surgical treatment, at least in cluster headache and related syndromes exists [11-14]. It follows that surgical procedures should be considered with great caution because no reliable long term observational data are available and because they can induce a secondary chronic pain condition as trigeminal neuralgia and/or anaesthesia dolorosa. Technical progress has recently introduced the opportunity to use neurostimulation rather than ablative or destructive methods and it may be applied to virtually any neural structure, including spinal cord, deep brain structures, motor cortex and peripheral nerves. It is not known how electrical stimulation of central or peripheral target structures exerts its effects, although a neuronal functional block seems the most likely option.

Almost all the mentioned therapies for RCM and CCH require weeks to months of stimulation for a prophylactic effect to occur, suggesting neuronal plasticity as a possible mechanism, and only stimulation of the sphenopalatine ganglion in CCH has demonstrated an acute, abortive effect [15]. Predictors of effectiveness for all modes of neurostimulation still need to be identified and in the future, the least invasive and most effective strategy must be preferred as first-line therapy for intractable chronic headaches [16].

Likewise neurostimulation should only be considered in patients that have tried all first-line therapies recommended in European guidelines [3], and that clinicians need to follow international consensus on that matter [10,17].

The neuroanatomical targets for these techniques vary. The theoretical mechanisms, therefore, may vary depending on the location of stimulation. Invasive neuromodulatory procedures comprise stimulation of the central nervous system (hypothalamic deep brain stimulation (hDBS)) and spinal cord stimulation (SCS) and of the peripheral nerves (occipital nerve stimulation (ONS), sphenopalatine ganglion stimulation (SPG). Non-invasive variants comprise vagus nerve stimulation (VNS), transcutaneous electrical nerve stimulation (TENS), repetitive transcranial magnetic stimulation (rTMS) and transcranial direct current stimulation (tDCS).

We aim to provide expert recommendations on the basis of a detailed review the present literature, a summary of clinical expertise and present a position for standard of care in the use of neuromodulation in chronic primary headaches in Europe.

## Methods

This review represents the view of the European Headache Federation (EHF) on this topic. The members of the Expert Group on Neuromodulation of chronic headache were appointed on the basis of their specific expertise on the topic with the necessary multidisciplinary approach.

All currently existing methods of neuromodulation have been reviewed and analysed if at least two case series have been published and the indications and limits of each of these methods are presented. Details of the ethical considerations and various study approvals are provided in the background literature, please see the reference list. Because the field is fast evolving and because neurostimulation has the intrinsic and principal challenge of unavailable placebo or sham conditions, this recommendation cannot strictly follow evidence based methods approaches. However, a modified Delphi conference mainly using Internet facilities has been used and all participants agreed to the recommendations presented here. This paper is therefore not a conventional guideline but international expert recommendations strictly based on published evidence.

## Results and discussion

### Hypothalamic stimulation

#### Theoretical background

Hypothalamic stimulation for drug-refractory CCH became a therapeutical target after PET studies showed increased blood flow in the posterior hypothalamus during cluster headache attacks [18], which was interpreted as neuronal activation of that brain area. A year later structural changes in the same brain area was demonstrated [19].

### Clinical data

In 2000, soon after these seminal studies, the first hypothalamic implantation and stimulation for drug-refractory CCH (dCCH) was performed [20] with favourable results. So far, data on more than 60 hypothalamic implanted patients are archived in the literature and include cluster headache patients and other types of trigeminal autonomic cephalalgia [20-39]. The overall success rate (patients pain-free or with ≥50% improvement) is around 50-60% and accumulated follow-up has made it possible to better understand advantages and limitations of the procedure.

The largest series to date comprises 19 severe dCCH patients [33]: after a mean follow-up of 8.7 years, long-lasting improvement was present in 71% (12/17) with 6 persistently almost pain free and another 6 no longer experiencing daily attacks but episodic attacks interspersed with long-lasting remission [33]. The pain free state was maintained after the stimulators had been off for a median of 3 years (range 3–4) in 5 patients, but this only happened after several years of continuous stimulation [33]. Most patients have headache recurrence a short time after the stimulator is switched off, or the battery runs out [20-39]. Five patients did not have benefit; 4 of these had bilateral cluster headache. Three of the non-responders experienced relief for the first 1–2 years but then developed tolerance [33]. Adverse events were electrode displacement (N=2), infection (electrode N=3; generator N=1), electrode mal-positioning (N=1), transient non-symptomatic 3rd ventricle haemorrhage (N=1), persistent slight muscle weakness on one side (N=1), and a seizure (N=1) [33]. Smaller studies have reported similar efficacy [20-39].

Eleven drug-resistant CCH patients were randomized to effective vs. sham posterior hypothalamic stimulation. No difference was detected between the two arms after one month, probably in relation to the short duration of treatment [30]. In the subsequent open-label phase, all patients received openly verum stimulation and three patients became pain free, and three others had a ≥50% reduction in attack frequency after 10 months.

Posterior hypothalamic activation has also been shown to be effective in three patients with short-lasting neuralgiform headache attacks with conjunctival injection and tearing (SUNCT), a rare form of trigeminal autonomic cephalgia. [40-42] The first patient became pain-free but additional prophylaxis with lamotrigine was necessary [40]. Another patient had a clinically significant reduction in attack frequency (from 120 to 25/day after a year) [41]. In the third patient [42] attack frequency dropped from 30/day to sporadic attacks after 15 months of continuous stimulation. A patient with chronic paroxysmal hemicrania also obtained relief after posterior hypothalamic stimulation [43].

Posterior hypothalamic stimulation has also been tested to abort acute cluster headache attacks. Treatment consisted of switching the stimulator on or increasing stimulation intensity. One hundred eight attacks were assessable and a ≥50% reduction in pain intensity was reported only in 23%; it was concluded that DBS is not useful for acute treatment of cluster headache [39].

### Safety and adverse effects

Overall, posterior hypothalamic stimulation is well tolerated years after implantation, but is not without risk: one patient died of intracerebral haemorrhage [21] and another had a subclinical 3rd ventricle haemorrhage [22]. In movement disorders, deep brain stimulation carries about a 3% risk of brain haemorrhage [40]. To reduce this risk Seijo et al. slightly shifted the hypothalamic target laterally so that the electrode tip was further from the lateral ventricle wall, without changing efficacy [32]. In line with this observation, a neuroimaging study showed that the anatomical location of the stimulating electrodes did not differ significantly between responders and non-responders [41]. Panic attacks [22], oculomotor disturbances, intraoperative transient ischaemic attack, subcutaneous infection, transient loss of consciousness with hemiparesis and micturition syncope, erectile dysfunction, and paroxysmal sneezing [20-33,42] have also been reported. Heart rate, blood pressure, and respiratory rate are not affected by hypothalamic stimulation when amplitude is increased slowly; however, sudden increase in amplitude can provoke autonomic and oculomotor disturbances [43]. Quality of sleep is improved during hypothalamic stimulation, possibly because of the suppression of nocturnal cluster headache attacks [44].

### Technical considerations

The first attempt to treat CCH by neuromodulation procedures was based on neuroimaging and particularly on the observation that a discrete volume of the posterior hypothalamus was activated during the pain bouts in CCH patients. The target of the procedure was the alleged hyperactive posterior hypothalamus (pHyp) and its inhibition was obtained delivering “in situ” high frequency current (180 Hz, 1–3 V, 60–90 μs pulse width) trough deep implanted electrodes.

### Limitations and recommendation for future studies

DBS is an invasive, expensive and probably non-specific technique that must be employed with caution and only carefully considered for the most severely affected patients with medically refractive CCH when other less invasive strategies have been employed. The hypothesis leading to the introduction of hypothalamic stimulation as a treatment for CCH was that high frequency electrode stimulation could reduce hypothalamic activation during a headache attack [12]. After long-term experience with the technique, it is now evident that this hypothesis is not correct: in fact acute hypothalamic stimulation does not abort acute cluster headache attacks [33], and it takes time – latency – for a prophylactic effect to develop, comparable to the delay in dystonia [20-33]. Taken together these observations indicate that stimulation works by a more complex mechanism, possibly brain plasticity [33,45-48].

### Occipital nerve stimulation (ONS)

#### Theoretical background

The rationale for the use of occipital nerve stimulation (ONS) in headaches came from animal studies showing the convergence of cervical, somatic and dural afferents on second order nociceptors in the trigeminocervical complex [49,50]. That suboccipital steroid injections turned out to be effective in the prevention of several primary headaches [51-53] was in favour of the existence of these anatomical connexions in humans. More than a decade ago, Weiner and Reed had already treated patients suffering from “occipital neuralgia” with ONS [54]. Their work paved the way for the use of this less invasive method of neurostimulation in various chronic headache types, essentially CCH and CM.

### Clinical data

Up to now 3 randomized sham-controlled (RCTs) ONS trials have been performed in CM [55-57] and their outcome is overall disappointing. The evaluation period was set at 12 weeks of ONS treatment in all of them. In the PRISM trial [55], available in abstract form only, 125 drug-refractory CM patients were treated with ONS or sham without any significant improvement. In the ONSTIM trial [56], 39% of patients (N=66) treated with active ONS during 3 months had at least 50% reduction in headache frequency and/or a 3-point intensity scale decrease, while there was no improvement in the non-stimulated or ineffectively stimulated groups. Finally, in a recent trial on 157 patients [57], the percentage of responders did not differ between active (17.1%) and control (13.5%) groups (primary endpoint). However, the number of headache days was significantly reduced in the ONS group compared to the sham population (−27.2% vs. -14.9%). The migraine-related disability also decreased with ONS. The main issue of this study is that patients were definitely not blinded to ONS (see below). Other existing studies of ONS in CM are small open trials or case reports (see [58] for review). Interestingly, the combination of occipital and supraorbital neurostimulation in an uncontrolled series of 7 CM patients [59] produced a ≥90% headache frequency improvement in all patients, while there was no significant response to either stimulation alone.

ONS has also been used in dCCH, but only open studies have been performed and in smaller groups of patients compared to the CM series. In the 3 main trials (13–15 patients), the success rate was slightly superior to 60% [58]. Burns et al. reported that after an average of 17.5 months under ONS therapy, 10/14 CCH patients were clinically improved: 3 had an improvement ≥90%, 3 a moderate amelioration (40-60%) and 4 a mild improvement (20-30%) [60]. In another study, 15 drCCH patients were prospectively followed up to 5 years after ONS implantation (mean 36.8 months) [61,62]. One patient was not evaluable due to an immediate device infection. Among the 14 remaining patients nearly 80% had a ≥90% reduction in attack frequency and 60% remained pain-free during long time periods (months to years). In another recent prospective trial (N=13, [63]) attack frequency decreased on average by 68% and intensity improved by 49%. Eight out of 13 patients were able to reduce or stop their preventive medications. Other smaller studies also report beneficial outcome of CCH patients under ONS (see [58] for review).

As far as other chronic forms of primary headaches are concerned, Burns et al. performed ONS in 6 patients with hemicrania continua (6–21 months [64]), and reported that 4 of them had a pain reduction exceeding 80%. Nine patients with drug-resistant SUNCT and 3 with SUNA (short-lasting unilateral neuralgiform headache attacks with conjunctival injection and tearing – SUNCT- or with autonomic symptoms - SUNA) had a benefit of at least 50% under ONS and 4 patients were nearly pain free after +/− 14 months follow-up [65,66].

### Safety and adverse effects

ONS is relatively safe compared to other invasive techniques, chiefly hypothalamic deep brain stimulation. The most frequent adverse events are lead migration, local immediate or delayed infections and battery depletion due to high stimulation intensities needed to obtain an optimal nerve stimulation in some patients (64% in [61]). Patients also complain of unpleasant traction on the connecting cables and sometimes do not tolerate the paraesthesias induced by the stimulation of the occipital nerves. Patients received generally bilateral ONS implantation even in side-locked headache forms, and in the only unilateral ONS series (in CCH) a headache side-shift was reported in 36% of them [61,62]. Bilateral ONS is therefore recommended.

ONS induces paraesthesias, like every other peripheral nerve stimulation. In our experience, the feeling of paraesthesias (covering the great occipital nerve or GON territory) appears mandatory to obtain a clinical improvement in CCH patients treated with ONS [61], but this is not always the case. Patients who do not feel the paraesthesias anymore (because of lead migration or battery depletion) often describe a recurrence of their headache attacks within the following days. There are no data demonstrating that ONS efficacy is conditioned by the stimulation of the GON or of the lesser occipital nerve or both, or correlated to the size of the area covered by paraesthesias. This phenomenon points out the main issue of ONS RCTs in headaches, i.e. the blinding. In CCH all available ONS studies are open trials and a placebo effect cannot be ruled out, even if in most patients attacks quickly relapsed after the stimulator was switched off. In CM more valid data are available and the outcome of the above mentioned RCTs is rather disappointing. More studies predicting a possible effect of ONS and patient selection are clearly warranted.

Few studies have been performed to understand ONS mechanisms in chronic headaches, and they suggested that ONS had a nonspecific neuromodulatory effect on central pain control systems. Hence, 36% of CCH patients successfully treated with ONS had still autonomic attacks despite the disappearance of the pain itself [61]. An 18FDG positron emission tomography (PET) study in 10 ONS-treated CCH patients showed an ipsilateral hypothalamic hyperactivity that remained unchanged during ONS therapy, contrary to the activity in pain transmitting cortical networks which normalized under ONS [66,67]. Similar modifications were also reported with activation PET in CM patients treated with ONS [68]. One could speculate that the ONS stimulation has an effect on the peripheral pain transmission but not on the central modulating areas.

### Technical considerations

There are many different stimulation electrodes but no comparative studies. The electrodes have to cross the GON in its subcutaneous course. Despite a great inter-individual anatomical variability, the GON becomes superficial approximately 1 cm below the occiput and 2–4 cm from the midline [69]. Consequently electrodes should ideally cover this spot. The electrodes have to be implanted subcutaneously above the fascia and always above the GON, which exhibit great anatomical variability [69]. As electrode migration is the most frequent complication, the leads have to be anchored firmly to the epifascial plane. Performing loops with the leads is recommended to allow extension of the leads during movements. Bilateral stimulation is recommended to avoid headache side-shift [61,62]. Implantation of the generator in the buttock is not recommended because the risk of migration could be higher. The release of flexible cables, epifascial anchoring and rechargeable batteries should decrease the cervical discomfort, lead migrations and battery depletion problems [58].

### Limitations and recommendation for future studies

ONS is an invasive, expensive and probably non-specific technique that must be employed with caution and only carefully considered for the most severely affected patients with medically refractive CCH. ONS demonstrated only preventive but no acute effect, with the exception of some chronic migraine patients [68]. Upcoming studies should be prospective, introduce a proper control and take the technical ONS challenges such as lead migration, frequent infections and proper blinding procedures into account. The mode of action is still speculative and the scientific evidence for a long lasting efficacy is lacking [70].

### Stimulation of the sphenopalatine ganglion (SPG)

#### Theoretical background

Strictly half-sided trigeminal pain along with parasympathetic activation is a central diagnostic feature of all trigeminal autonomic cephalgias (TAC’s) [71]. Consequently, several studies have targeted the facial parasympathetic output by blocking [72,73] or lesioning [74] the sphenopalatine ganglion (SPG). The SPG is a large extracranial parasympathetic ganglion located in the pterygopalatine fossa (PPF). Post-ganglionic parasympathetic fibers from the SPG innervate facial structures such as the salivary and lacrimal glands, the nasopharyngeal mucosa and the cerebral and meningeal blood vessels [75]. Because cluster headache is such a vicious pain which is not always medically treatable [9], various invasive interventions in the PPF have been tried including alcohol injection, thermocoagulation [76], transnasal injection of lidocaine [73], neuroablation [77], radiofrequency lesions [78] and pulsed radiofrequency ablations [74]. The success rates seem promising (varying from 46 to 85%), but the benefits have been transient [79]. Because of this transient nature and because of the irreparable side effects of the lesioning interventions, a non-destructive approach using acute percutaneous SPGS with a removable electrode was examined in five patients with cluster headache. This small pilot study showed a success rate of 61% [80], which led to another pilot study in patients with acute migraine attacks which also showed some efficacy [81].

### Clinical data

Based on these findings, a new kind of implantable microstimulator in the facial region was developed and a multicenter randomised double-blind and sham-controlled trial has been conducted to examine the efficacy of acute stimulation in refractory CCH. This device is powered and controlled transcutaneously by electromagnetic waves [15]. In this study, 68% of the 32 enrolled CCH patients benefited from electrical stimulation of the SPG [15]. Surprisingly, patients showed two positive effects: full stimulation of the SPG versus sham stimulation resulted in a significant pain relief (which was the main outcome parameter) and a significant reduction in attack frequency. The pain relief and pain freedom rates at 15 minutes were 67% and 34% respectively and significantly greater than with subthreshold or placebo stimulation. It needs to be pointed out that this study cannot answer the question how long these effects will continue but the impression at the moment is that these effects last and long term follow up studies are underway. The surprising observation that there was a significant reduction of headache attack frequency in addition to the acute response has to be seen with caution, as this study was designed and powered to test the acute effects on spontaneous cluster headache attacks. Overall, 43% of patients experienced an attack frequency reduction of ≥ 50% from baseline, which is remarkable as all patients had been suffering from the CCH for many years and had tried a number of preventive drugs without benefit. Given the slight tingling sensation that is accompanying stimulation of the SPG, a placebo effect cannot be excluded but the apparent preventive effects of SPG stimulation certainly warrant further investigation.

### Safety and adverse effects

Of note, oral maxillofacial surgeries are inherently associated with standard peri-operative adverse events, including pain, swelling, hematoma, infections and sensory disturbances. While the rate of device-related complications was however quite low, sensory disturbance (81% of patients) and pain (38%) were the most frequent side-effects immediately after the implantation, mainly affecting maxillary nerve branches. However, after 3 months, only 16% of patients suffered from ongoing and mild sensory disturbance and 19% from local pain, respectively [20]. No other significant neurological side effects were observed. In summary, local sensory impairment seems to be a mild complication compared to the severe cluster attacks but the implantation procedure needs further attention. Overall, SPG stimulation appears to rank among the minimally invasive and safe neuromodulatory strategies.

### Technical considerations

Implantation of the ATI-SPG-Stimulator is done under general anesthesia via a vestibular incision of the posterior maxillary mucosa of the affected side (trans-oral, gingival buccal technique). The stimulating electrodes on the integral lead are positioned within the PPF proximate to the SPG, with the body of the SPG Neurostimulator positioned on the lateral-posterior maxilla medial to the zygoma and anchored to the zygomatic process of the maxilla using the integral fixation plate. After implantation, positioning control is confirmed by doing a three-dimensional imaging (parasinus CT) of the PPF. Patients then undergo a therapy titration period during which stimulation parameters are to be adjusted bi-weekly. Individual electrical stimulation parameters are adjusted according to provoked paresthesias in the root of the nose and/or treatment effect during an attack. The maximum amplitude is usually programmed to be slightly higher than the amplitude that provoked discomfort in each patient. If neurostimulator lead positioning is determined to be incorrect, a lead revision procedure should be considered.

### Limitations and recommendation for future studies

Judging from the published data, the input of the parasympathetic system in the origination of cluster headache attacks is significant. This is underlined by a recent report that low-frequency SPG stimulation can provoke attacks in patients with cluster headache which in turn can be treated with high-frequency stimulation [82]. It has to be kept in mind that all of the above data have been reported in medically intractable patients with CCH. It may be worthwhile using the method of SPG stimulation in episodic cluster headache patients, however, given the above mentioned side effects, only in patients with particularly long active bouts and failure of preventative medication. Given that only one placebo-controlled study exists to date, this method should be still seen as experimental until further studies are presented.

### Vagal nerve stimulation (VNS)

#### Theoretical background

The first investigations on the modulation of nociception by vagal afferents were performed approximately 20 years ago [83-85]. In animals it has been demonstrated that electrical, chemical, and physiologic activation of vagal afferents produces analgesic effect [86-91]. The activation of vagal afferents decreases the activity of second order nociceptive neurons in the spinothalamic and spinoreticular tract of the spinal cord [84,88,92] – resulting in inhibition of spinal nociceptive reflexes and spinal nociceptive transmission [87,92] – and in the trigeminal nuclear complex [93-95].

### Clinical data

Only smaller open case series exist. In a retrospective survey, three of four patients with implanted VNS reported a substantial improvement of migraine frequency and pain scores [96]. One of the 4 patients became migraine-free 1 month after the onset of VNS. A second patient had a reduction of >50% in both frequency and severity. A third patient reported >50% reduction in frequency. The final patient had a slight reduction in both frequency and severity. Improvement was reported to start 1 to 3 months after initiation of therapy. In another retrospective study, eight of ten patients with migraine had a 50% or more reduction in headache frequency, with five of them completely headache free in the 6 months after treatment initiation, with improvement occurring in the first 3 months following stimulator placement [97].

A case series reported a good response to VNS in two of four patients with chronic migraine (one with a subdiagnose of basilar-type migraine (BTM) and hemiplegic migraine (HM) and the other with BTM) and in two patients with CCH [98]. Recently, a novel method has been described to non-invasively stimulate brain structures in a similar way to VNS [99-101]. The method is based on the technique of transcutaneous electrical nerve stimulation (TENS), which is used in acute and chronic pain syndromes. t-VNS is delivered by a medical device to the left auricular branch of the vagus nerve (t-VNS) located medial of the tragus at the entry of the acoustic meatus without any surgery. Another novel method is also thought to stimulate the vagus nerve transcutaneously (tVNS). Preliminary data suggested that tVNS could be effective in selected patients [102]. In a pilot trial evaluating 13 primary headache sufferers, however, ten stopped tVNS because lack of efficacy and/or side effects [103].

### Safety and adverse effects

The very limited experience with both implantable and transcutaneous VNS prohibits a clear presentation of safety and limitations in use. Based on the experience of VNS in medically intractable epilepsy the method seems fairly safe and mainly hampered by infections and battery problems. The reported adverse effects are mainly transient muscle cramps and local pain, which can be reduced by the applied stimulation paradigm. So far, no significant safety issues have been raised but clinical experience is very scarce.

### Technical considerations

VNS sends electrical signals along the part of the vagus nerve that runs through the neck. Data suggests that VNS reduces the amount of glutamate, substance associated with headache symptoms, in the brain.

The VNS therapy is administered with a hand held device, placed on the neck, which produces a mild electrical signal transmitted to the vagus nerve through the skin.

It is possible to turn up the stimulation strength until the patient feels a mild sensation underneath the skin. The duration of each treatment is approximately 2 minutes.

### Limitations and recommendation for future studies

Considering the small series of patients studied, no firm conclusion can be drawn. Until proper evidence is provided devices claiming to stimulate the vagus nerve transcutaneously should be preferred to more invasive techniques. Due to the lack of evidence, VNS should only be employed in chronic headache sufferers using a randomized, placebo controlled trial design. Currently some RCTs are ongoing to validate this therapeutic approach to chronic headaches (NCT01667250, NCT01701245).

### Transcranial direct current stimulation (tDCS)

#### Theoretical background

Recent progress in transcranial neurostimulation techniques has been used to approach the treatment of chronic therapy resistant headache. In particular transcranial direct current stimulation (tDCS) applied through the skull has been shown to directly modulate the excitability of cortical areas, best investigated for human motor (for a review see: [104]) and visual (for a review see: [105]) cortices. tDCS induces both acute and persistent neuronal excitability changes in the cortex, probably by shifting neuronal resting membrane potential and hereby modulating the spontaneous discharge rates of cortical neurons [106-109]. The after-effects of tDCS are most easily studied at the primary motor cortex (M1) by measuring the amplitude changes of the motor evoked potentials (MEP) using transcranial magnetic stimulation (TMS) [110]. A minimal duration of 3 minutes and at least 0.4 mA stimulation intensity is necessary to induce cortical excitability changes outlasting the stimulation duration [110,111]. At rest, cathodal stimulation induces a decrease and anodal stimulation an increase of cortical excitability. The effect of tDCS origins intracortically; pharmacological studies have shown that the effects during stimulation are mediated by ion-channels, in accordance with a primary hyper- or depolarizing effect of the stimulation, while after-effects involve the modulation of N-methyl-D-aspartate- (NMDA) receptor efficacy [112].

### Clinical data

Using tDCS as a treatment for chronic headaches only data on treatment of orofacial pain [113] and migraine are available. Based on a concept of cortical hyperexcitability in migraine cathodal tDCS in migraineurs is expected to normalize the cortical excitability either (i) by prophylactic treatment in the interictal phase or (ii) by an acute treatment at the beginning of the migraine attack. So far three studies evaluated the effect of repeated application of tDCS as a prophylactic treatment. Antal et al. [114] has investigated cathodal stimulation of the primary visual cortex (V1). 30 patients were randomly assigned to cathodal or to sham stimulation. 26 patients participated in the final analyses (cathodal: 13, sham: 13). Compared to the sham group, only the intensity of the pain was significantly reduced after verum stimulation.

Auvichayapat [115] and coworkers have investigated 42 episodic migraine patients, that were randomized to receive either active or sham stimulation on a daily basis for 20 consecutive days. The results showed statistically significant reduction in attack frequency and abortive medications at week 4 and 8 after treatment. The pain intensity was statistically significant reduced at week 4, 8, and 12.

In the third study [116] thirteen patients with CM were randomized to receive 10 sessions of anodal (n=8) or sham (n=5) tDCS for 20 minutes over 4 weeks. There was a significant interaction term for the pain intensity and for the length of migraine episodes. Post-hoc analysis showed a significant improvement in the follow-up period for the active tDCS group only (delayed response).

Phase III studies are still missing as well as data in the acute migraine phase or at the beginning of the aura. Similarly, there are no data available concerning other type of headaches, such as cluster and tension- type headache.

### Safety and adverse effects

Amongst transcranial stimulation device-based interventions, tDCS is generally considered to be easier to blind than TMS [117]. The type of stimulation cannot be judged by an outside observer and it is easily applicable. By far the most widely reported phenomenon associated with the application of both active and sham tDCS stimulation is the itching or tingling sensation under the electrode [108,114,118]. Other, less frequently reported phenomena associated with the stimulation are burning sensations, headache, redness of skin, nausea and light flashes at the beginning and the end of the stimulation [119]. It was recently reported that cutaneous perception does not completely disappear in the first phase of the stimulation as previously reported but never quantitatively assessed [119]. Nevertheless, in naive and even in experienced participants, no significant differences in the levels of perceived stimulation strength could be observed between sham and verum stimulation, thus the ramping up – short stimulation (30 sec) – ramping down method might be a reliable approach to blinding in tDCS research, at least when using stimulation intensity below 1 mA [119].

### Technical considerations

Aftereffects of tDCS are NMDA receptor dependent [112]. Patients on NMDA receptor antagonists, e.g. on dextromethorphan, an anticoughing drug, might not benefit from both anodal and cathodal tDCS. Sodium channel blocking agents such as carbamazepine and calcium channel blocking agents selectively prevent anodal tDCS aftereffects [120]. Flunarizine as a calcium antagonist is used in some countries for migraine prophylaxis. Also propranolol shortens both cathodal and anodal aftereffects [121]. Rarely safety issues play a role [119,122]; e.g. no metal should be implanted in the head. Precautious exclusion of patients with previous history of brain surgery is warranted due to higher current density if the electrode is closer than 2 cm to a skull deficit. Neurological disorders such as stroke or epilepsy, drug/alcohol dependence, major psychiatric co-morbidities and implanted pacemaker may be seen as an exclusion criterion. There is probably no risk for women in child-bearing age without contraception, women during pregnancy and lactation to be expected, if both electrodes are fixed at the skull, however no data exist on that. It would be prudent to exclude this group from stimulation.

### Limitations and recommendation for future studies

Cathodal V1 stimulation pursues the concept of inhibition of a hyperexcitable visual cortex [114] whereas anodal M1 stimulation pursues the concept of M1 excitation for reduction of pain perception [115,116]. Stimulation protocols will be further optimized in future. Repeated applications of the stimulation are probably necessary, testing different intensities and stimulation paradigms. For practical use and for longer lasting studies stimulators that can be used at home should be available.

It appears to be mandatory for controlled studies that the subjects or patients are asked after the stimulation if they believe to be in the verum or placebo group. Similarly, it is important to document the expectation of the patients with regard to the stimulation outcome in order to be able to better estimate placebo effects. RCT’s on other chronic primary headaches are also warranted.

### Repetitive transcranial magnetic stimulation (rTMS)

#### Theoretical background

Introduced by Barker et al. [123], transcranial magnetic stimulation (TMS) is a neurostimulation tool able to perform painless cerebral stimulation through application of magnetic fields on the scalp. The magnetic current passes through the scalp and generates a perpendicular electrical current that flows tangentially to cortex generating action potentials in cortical neurons. If given in repeated pulses, rTMS can determine long lasting plastic effects that remain also after the end of the train and depend on the stimulation frequency used: frequencies ≤1 Hz (low-frequency rTMS: LF-rTMS) reducing, while frequencies >1 Hz (high-frequency rTMS: HF-rTMS) increasing cortical excitability [124,125].

TMS has been employed in two different ways in migraine, either to treat the single attack or prevent its occurrence. Different approaches were done, in consideration of mechanisms subtending the occurrence of migraine and the development into chronic form [126-130].

### Clinical data

Single pulse trans-cranial magnetic stimulation of the occipital cortex, was employed by a portable apparatus, to be tested in migraine with aura attacks in a double blind sham controlled study, involving a total of 164 patients and showed a significant effect of verum over sham treatment [131]. Brighina et al. first [132] evaluated the efficacy and tolerability of HF-rTMS over the left Dorsolateral Prefrontal Cortex (DLPFC), (an area known for its top-down control on nociceptive transmission [133]) for preventive treatment in patients affected by chronic refractory migraine. Patients were randomly assigned to active, real (6 patients) or placebo sham (5 patients) rTMS treatment consisting of 12 stimulation session delivered on alternate days. As compared to baseline and sham rTMS, active treatment reduced migraine attacks (about 57% less), drug consumption, headache index, and migraine disability scores) in the month during and following stimulation. Misra et al. [134] used HF-rTMS of motor cortex (another area able to exert control on pain mechanisms [135]) for prophylactic treatment in patients with episodic and chronic migraine; authors explored also the relationship between migraine pain and β endorphin plasma levels. The results demonstrated the ability of M1 rTMS to significantly reducing headache frequency (about 85% less at 1^st^ week after stimulation), headache severity, functional disability and analgesic intake.

### Safety and side effects

TMS and rTMS are generally well tolerated and safe as only minor side effects like transient mild headache or local pain and paresthesias are reported [136]. However, the procedure is to be avoided in patient with skull defect or with pacemaker, cardiac lines, metal in the head (electrodes, stimulation devices) or other apparatus that could be influenced (dislocation, induction of electric currents) by magnetic field. Caution should be paid in patient with epilepsy, because a risk (even if really low!) for seizure is reported. No side effect has been reported in pregnant women treated with HF-rTMS for refractory depression, though [137]; however, giving the lack of enough evidence, rTMS is not recommended in such condition [137].

### Technical considerations

Paradoxical effects to rTMS (facilitation to inhibitory LF-rTMS or decremental response to facilitatory HF-rTMS) has been reported in patient with migraine [138-141]. Moreover, effects of rTMS can be consistently modulated (influenced) by several drugs (expecially antiepileptics like topiramate and valproate) employed in migraine prophylaxis. These factors should be taken into account when planning and/or interpreting results of stimulation trials.

### Limitations and recommendation for future studies

Considering the few trials performed and the small series of patients studied, no firm conclusion can be drawn by these studies and it is uncertain whether the effect is acute, preventive or both. rTMS appears to be a safe [136] and potentially effective tool for treatment of chronic migraine patients who showed resistance to pharmacological treatments [58]. Further studies are needed to assess factors underlying therapeutic effects (change in cortical excitability, better antinociceptive control, both?). It’s also to seek for optimal stimulation parameters (intensity, frequency, number and duration of stimulation sessions). Another important point may be the best cortical areas to be modulated for pain control in migraine, and the most efficacy side of stimulation, though the left side has been more frequently employed in studies on pain control [142]. Particularly useful would be the generation of stimulation devices that patients can use at home.

### Transcutaneous stimulation of cranial nerves and TENS

#### Theoretical background

Transcutaneous electrical stimulation techniques have a long tradition in chronic pain management. These techniques are rather inexpensive and non-invasive, but the evidence for their effectiveness is overall of low quality [143]. There are limited data on the use of electric current to stimulate cutaneous nerves (transcutaneous electrical nerve stimulation or TENS) or specific cranial nerves (supraorbital and supratrochlear) nerve stimulation (tSNS) in the treatment of headache disorders. The restrictive definition of TENS is the administration by surface electrodes of electric current produced by a device to stimulate cutaneous sensory nerves to reduce pain, both acute and chronic. Indeed, TENS treatment targets painful regions (or acupoints in electroacupuncture) instead of specific nerves. Based on the stimulation frequency, TENS can be subdivided in low frequency (frequency <10 Hz) or high frequency (frequency >10 Hz). As the biological basis of analgesia by TENS remains speculative, the ‘gate control theory’ of pain is the most tenable explanation but release of endogenous opiates could be involved [144].

### Clinical data TENS and tSNS

#### TENS

Several meta-analyses on the efficacy of TENS in painful disorders have yielded ambiguous or negative results mainly due to inadequate methodology and/or reporting [143,145-148]. TENS treatment for headache disorders appeared in the literature as early as 1975 [149]. Acute effects of TENS have been suggested in a study from Solomon and Guglielmo published in 1985. Sixty-two patients with migraine or “muscle contraction headache”, who experienced a headache at the time of their visit, were divided into 3 different groups receiving either full high frequency-low intensity TENS, subliminal stimulation or placebo stimulation for 15 min once resulting in a significant but usually slight to moderate improvement in pain severity immediately after the intervention [150]. A Cochrane review from 2004 concluded that the use of TENS for chronic/recurrent headache (including migraine, tension-type headache, cervicogenic headache and post-traumatic headache) prophylaxis is not supported by conclusive evidence [151], and ever since very little original trial data have been generated. In a recent trial, the efficacy of intermittent low frequency-high intensity TENS administered to the temporal and occipital region for a total of 10 weeks was compared to the preventative effect of 50 mg imipramine per day for 3 months in a sample of 138 patients with chronic tension-type headache [152]. After 3 months compared to the baseline, the headache intensity on the VAS score showed a significant decrease in both approaches with a numerically higher reduction in the imipramine group. Although the sample size was relatively large, a placebo arm was not included and use of the VAS score as a primary outcome is questionable.

#### tSNS

A recent Belgian multi-centric randomized controlled trial on the efficacy of transcutaneous supraorbital (and supratrochlear) nerve stimulation (tSNS) in episodic migraine, the PREMICE study, included 67 patients in the final analysis [153]. A significant decrease of 2.06 headache days per month was observed in the group receiving full stimulation (p=0.023) compared with only 0.32 days in the sham group (p=0.608) [153]. The comparison between both groups missed significance by a narrow margin (p=0.054). The 50% responder rate was significantly higher in the verum (38.1%) than in the sham (12.1%) group (p=0.023). However, the observed effects were only moderate and despite a number of precautions by the investigators unblinding may have occurred as effective stimulation induces marked paresthesias [153]. Therefore, assessment of unblinding should be mandatory for future neurostimulation studies.

### Safety and side effects

High frequency TENS delivered at low intensities is associated with paraesthesia over the area of stimulation, and low frequency TENS delivered at high intensities is associated with a sharp flicking sensation or even muscle contractions. These sensations hamper proper blinding in controlled trials.

### Technical considerations

Effective blinding with feasible sham paradigms is still an unresolved issue in transcutaneous stimulation of cranial nerves and TENS making large-scale studies difficult. In addition, stimulation parameters differ widely in TENS studies and consensus settings for clinical studies are missing.

### Limitations and recommendation for future studies

The methodology in headache studies differs profoundly and a convincing sham paradigm has not been established. At present there is insufficient evidence for the use of TENS in headache prophylaxis and to abort an acute headache.

Lack of evidence of effect is however different from evidence of lack of effect [143]. So far, a single study provided Class III evidence that migraine attacks can be prevented with tSNS, but the effect size was small, unblinding may have occurred. The effects of tSNS on very frequent or chronic migraine are unknown, and refractory patients were excluded. Widespread use outside of controlled studies of this potentially valuable treatment modality cannot be endorsed at present [153].

### Spinal cord stimulation

#### Theoretical background

The Occipital Nerve Stimulation (ONS) technique takes advantage of the “functional overlap” of the higher cervical roots and the trigeminal nucleus to neuromodulate, in a retrograde fashion, the trigemino-cervical complex [154,155]. However, it is a reasonable assumption that the application of electrical pulses directly onto the dorsal columns at the C2-C3 vertebral level will provide a neuromodulatory effect on the TCC similar to – if not greater than – peripheral occipital nerves stimulation. Cervico-medullary spinal cord stimulation has been used for the last 30 years to alleviate intractable head and facial pain [156], but it requires a very costly, time consuming and complex neurosurgical procedure and the mode of action is unknown.

### Clinical data

Performed in few specialised centres and in highly selected patients, and never in a controlled design, it is not a viable option for primary headaches. Recently, low-frequency stimulation of the cervical spinal cord (C2-C3 level) has shown positive results in a case series of CCH patients implanted with a percutaneous cervical epidural lead [157]. Authors reported a marked reduction in headache’s frequency (−4.6 attacks per day), intensity (−2.9 on a VAS score) and duration (−27 minutes per attack) in 7 patients implanted. The procedure also facilitates a 4–19 days testing phase prior to permanent implant. However, this study was criticized [158].

### Safety and adverse effects

Adverse effects such as lead migration, battery depletion and local infections are inherent in neuromodulatory approaches and have been reported in hypothalamic brain stimulation [20-33,42], occipital nerve stimulation [61,62,68], and stimulation of the sphenoid ganglion [21]. However, the rate reported in SCS of the cervical region seems exceedingly high [157,158] and resulted in repetitive invasive procedures, mostly lead revision. Given that a dislocation of the lead is an inherent problem in spinal cord stimulation especially in parts of the spinal cord with high mobility such as the upper cervical spine, less invasive methods such as the occipital stimulation or SPG-stimulation should be preferred at least until ongoing studies (see below) are published.

### Technical considerations

Different stimulation frequencies are now available (burst stimulation, 10 kHz high frequency stimulation) in SCS. Those provide a new alternative to peripheral (low-frequency) stimulation due to their ability to achieve pain relief without causing any perceived sensation but its efficacy and potential side effects are unknown. A double-blind, placebo design can now be considered when planning future randomized control trials of SCS in chronic, refractory headaches.

### Limitations and recommendation for future studies

A “proof of concept” pilot study investigating the initial tolerability and efficacy of cervical high-frequency SCS in the treatment of refractory CM is under way (NCT01653340) and preliminary results are expected by the end of 2013. Until proper evidence is provided the present expert group recommends that spinal cord stimulation is strictly avoided in patients with primary headache syndromes.

## Conclusion and General Recommendation

The purpose of this position paper, as a result of the collaboration of an multidisciplinary Expert Group on Neurostimulation of the European Headache Federation, is to give an assessment and recommendation for the use of the currently available neuromodulation devices in headache treatment. This overview is based on the scientific level obtained through controlled studies, on existing clinical practice, directly related side effects and overall safety. Because the available data regarding the various stimulation approaches are so scarce and variable, this recommendation is also based on the definition of a clinically significant improvement. In 2008, recommendations put forth by the Initiative on Methods, Measurement, and Pain Assessment in Clinical Trials (IMMPACT) panel and the IHS established a 30% reduction in pain as clinically meaningful [31,32]. The authors of this recommendation feel that this minimal requirement is only sufficient in otherwise medically intractable chronic patients, otherwise a 50% reduction in pain should be acquired. Prevention of headache days is certainly the single most clinically relevant item in medical intractable patients and the most important reason why these patients seek therapy. However, clinical trial assessments should not be limited to the degree of pain relief or headache days because this alone may not be necessary for a clinically meaningful improvement, but should include tolerability, reductions in headache-related disability, improvement in pain-specific quality of life, total costs and improvement in functional capacity. Unfortunately, regarding these outcome parameters even less data exists for neurostimulation devices in headache treatment.

For all of the above mentioned methods and devices, the following recommendations are uniquely effective and have to be seen as the basic qualification and requirement which may be additional to the specific recommendations for each method as outlines in the respective chapter.

1) *From a medical standpoint, the application of a neurostimulator, either in a trial or on the basis of a CE mark treatment, should be considered only once all alternative drug and behavioural therapies as recommended by international guidelines have failed and medication overuse headache is excluded.*

2) *This involves that the patient is considered chronic, following the current IHS definition*[39]*and have been evaluated at a tertiary care headache center.*

3) *This involves that the patient is considered medically intractable as defined by international consensus*[10].

4) *Non-invasive medical technologies should be considered prior to implantation of a neurostimulator and the least invasive and most effective treatment should always be first line therapy.*

Given the heterogeneous data in terms of patient numbers, inclusion requirements, headache diagnosis, statistical methods and completeness of data in published studies, the authors cannot unequivocally give a ranking of neurostimulation methods. The global evaluation leads to the following *ad interim* conclusion:

1) *In CCH it is advisable to use SPG*[79,80]*or ONS*[55,59]*, before considering DBS. Although the treatment effects seem clinically equal, the side effects of the more invasive DBS treatment are to be considered*[43]

2) *In CM the use of ONS seems acceptable although based on limited evidence. Application of the non-invasive tVNS, tDCS, rTMS, TENS and tSNS in chronic headaches are not yet evidence based, given the poor amount of controlled data. However, it needs to be mentioned that these devices are relatively harmless when compared to more invasive and costly neurostimulation devices and may be tried before using more invasive neurostimulation devices.*

*The authors note that therapeutic neurostimulation* in headache and pain is a fast evolving field and that no recommendations can be given using the methodological arsenal of evidence based medicine. One of the reasons is the limited use of a proper placebo condition or sham control and randomized sham and subthreshold stimulation was included only in the SPG study on acute Cluster headache. While sham is in principle available in central neuromodulation (DBS) [30] it is nearly impossible in peripheral neuromodulation devices, given that peripheral nerve stimulation is always perceived. However, we recommend that proper done controlled and randomized studies are required before a given neurostimulation device is implemented and clinically used. A CE-mark is not equivalent to a randomized study following IHS requirements, as no clinical data supporting the benefit of a medical device are needed to acquire the CE mark, but only data showing that the respective device is probably harmless. The authors suggest the following recommendations for clinical trial involving neurostimulation devices in headache treatment:

1) *Trials investigating invasive neurostimulator devices should only involve patients who are considered chronic, following the current IHS definition*[38]*. If a given method proved efficacy in the chronic state, follow-up studies may broaden the indication to severely disabling episodic states, if medically not sufficiently treatable.*

2) *Trials investigating neurostimulator devices should only involve patients who are not suffering from medication overuse headache and are considered medically intractable as defined by international consensus*[10]*.*

3) *Clinical trial assessments should have the primary endpoint of the degree of pain relief or reduction in headache days. Next to adverse events, secondary endpoints should include reductions in headache-related disability, improvement in pain-specific quality of life and improvement in functional capacity.*

In summary, neurostimulation should only be considered in patients that have tried all first-line therapies recommended in European guidelines [3], and that both pain and headache clinicians need to follow international consensus on that matter [10,17]. The greatest limitation for clinical use is the lack of proper controlled studies [159]. Consequently, any devices that have not been investigated in such controlled studies and have shown to be effective with an acceptable side effect profile should not be used at all. The authors note that it is inherent to neurostimulation devices, perhaps with the only exception of DBS so far, to lack a proper placebo condition. Most available trials actually used infrathreshold stimulation intensities as controls, but blinding in patients perceiving no or few sensations may be difficult to maintain. It is crucial to recruit neurostimulation naïve patients for future trials, but as a recent editorial suggested this will be an increasing challenge due to the negative role of the social media (Internet blogs, Facebook etc.) [160]. International guidelines, preferably agreed between the IHS and EHF how to conduct such studies are clearly warranted.

## Abbreviations

EHF: European Headache Federation; LTB: Lifting The Burden; EFIC: European Federation of IASP Chapters; rCCH: Refractory chronic cluster headache; rCM: Refractory chronic migraine; hDBS: Hypothalamic deep brain stimulation; ONS: Occipital nerve stimulation; SPG: Stimulation of sphenopalatine ganglion; HF-SCS: High frequency spinal cord stimulation; VNS: Vagal nerve stimulation; tDCS: Transcranial direct current stimulation; rTMS: Repetitive transcranial magnetic stimulation; tSNS: Transcutaneous stimulation of cranial nerves; TENS: Transcutaneous electrical nerve stimulation.

## Competing interests

The following Authors declared competing interests related to the contents of this manuscript:

RA received travel grants and consulting fees from Medtronic, Boston Scientific, Nevro Corporation and St Jude Medical.DF received travel grants or consulting fees from Boston Scientific and St Jude Medical. RHJ have lectured for Pfizer, Berlin-Chemie, Allergan, Merck, has received consulting fees from Autonomic Technologies Inc, Medotech, and Linde Gas, is member of advisory boards of Autonomic Technologies Inc, Medotech, Neurocore, and Linde Gas. TPJ received honoraria as speaker and consulting fees from Autonomic Technologies, Inc. PM received travel grants, consulting fees or unrestricted grants from Nevro Corporation, St Jude Medical, Allergan, Pfizer, ACRAF, Springer, is member of Advisory Board in Allergan. AM is or has been consultant or speaker for Pfizer, Bayer Vital, GSK, Allergan, ATI, MSD, and Desitin. He has received unrestricted grant support from LindeGas and Almirall. KP has received a travel grant from Medtronic and a research grant provided by Autonomic Technologies Inc. SP received travel grants from Medtronic and Nevro Corp. WP received consulting fees from EBS Technologies.

Other Authors declared no competing interests.

## Authors’ contribution

All Authors are members of the Expert Group on Neurostimulation of Chronic Headaches of European Headache Federation. PM, AM and RHJ conceived the study, provided overall guidance and oversaw the implementation of the work. All Authors prepared the first draft on the basis of their specific scientific and clinical expertise: Hypothalamic stimulation (ML, AF); ONS (DM, DF); SPG (AM, MH); HFSCS (PM, RA, SP); VNS (PM, RHJ); tDCS (AA, WP); rTMS (FB, MdT); TENS and TSCN (TPJ, KP). PM, RHJ and AM finalized the draft based on comments from other authors and reviewer feedback. No external assistance in selection, writing or reviewing data has been utilized. RHJ is director and trustee in LTB, EHMTIC and President of EHF. PM is director and trustee in EHF and chairman and trustee in LTB, chairman of EFIC Joint Campaign against Headache (EFIC-EHF-LTB), president elect of Italian headache society. AM is vice president of the German headache society and director in IHS. All authors read and approved the final manuscript.

## References

[B1] Vos T Flaxman AD Naghavi M Lozano R Michaud C Ezzati M Shibuya K Salomon JA Abdalla S Aboyans V Abraham J Ackerman I Aggarwal R Ahn SY Ali MK Alvarado M Anderson HR Anderson LM Andrews KG Atkinson C Baddour LM Bahalim AN Barker-Collo S Barrero LH Bartels DH Basáñez MG Baxter A Bell ML Benjamin EJ Bennett D Years lived with disability (YLD) for 1160 sequelae of 289 diseases and injuries 1990–2010: a systematic analysis for the global burden of disease study 2010 Lancet 2012 380 2163 2196 23245607 10.1016/S0140-6736(12)61729-2 PMC6350784

[B2] Steiner TJ Stovner LJ Birbeck GL Migraine: the seventh disabler Headache Pain 2013 14 1 10.1186/1129-2377-14-1 PMC3606966 23566305

[B3] May A Leone M Afra J Linde M Sándor PS Evers S Goadsby PJ Task Force EFNS EFNS guidelines on the treatment of cluster headache and other trigeminal-autonomic cephalalgias Eur J Neurol 2006 13 1066 77 16987158 10.1111/j.1468-1331.2006.01566.x

[B4] Evers S Afra J Frese A Goadsby PJ Linde M May A Sándor PS European Federation of Neurological Societies EFNS guideline on the drug treatment of migraine--revised report of an EFNS task force Eur J Neurol 2009 16 968 981 doi: 10.1111/j.1468-1331.2009.02748.x 19708964 10.1111/j.1468-1331.2009.02748.x

[B5] Steiner TJ Antonaci F Jensen R Lainez MJ Lanteri-Minet M Valade D European Headache Federation, Global Campaign against Headache Recommendations for headache service organisation and delivery in Europe J Headache and Pain 2011 12 419 426 doi: 10.1007/s10194-011-0320-x 21380555 10.1007/s10194-011-0320-x PMC3139057

[B6] Lionetto L Negro A Palmisani S Gentile G Del Fiore MR Mercieri M Simmaco M Smith T Al-Kaisy A Arcioni R Martelletti P Emerging treatment for chronic migraine and refractory chronic migraine Expert Opin Emerg Drugs 2012 17 393 406 22862686 10.1517/14728214.2012.709846

[B7] Negro A Martelletti P Chronic migraine plus medication overuse headache: two entities or not? J Headache Pain 2011 12 593 601 21938457 10.1007/s10194-011-0388-3 PMC3208042

[B8] Schulman EA Peterlin BL Lake AE 3rd Lipton RB Hanlon A Siegel S Levin M Goadsby PJ Markley HG Defining Refractory Migraine: results of the RHSIS Survey of American Headache Society Members Headache 2009 49 509 518 19245385 10.1111/j.1526-4610.2009.01370.x

[B9] May A Cluster headache: pathogenesis, diagnosis, and management Lancet 2005 366 843 55 16139660 10.1016/S0140-6736(05)67217-0

[B10] Goadsby PJ Schoenen J Ferrari MD Silberstein SD Dodick D Towards a definition of intractable headache for use in clinical practice and trials Cephalalgia 2006 26 1168 70 16919073 10.1111/j.1468-2982.2006.01173.x

[B11] Matharu MS Goadsby PJ Persistence of attacks of cluster headache after trigeminal nerve root section Brain 2002 125 976 984 11960888 10.1093/brain/awf118

[B12] Black D Dodick DW Two cases of medically and surgically intractable SUNCT: a reason for caution and argument for a central mechanism Cephalalgia 2002 3 201 204 12047459 10.1046/j.1468-2982.2002.00348.x

[B13] Jarrar RG Black DF Dodick DW Davis DH Outcome of trigeminal nerve section in the treatment of chronic cluster headache Neurology 2003 60 1360 1362 12707445 10.1212/01.wnl.0000055902.23139.16

[B14] Donnet A Valade D Regis J Gamma knife treatment for refractory cluster headache: a prospective open trial J Neurol Neurosurg Psychiatry 2005 76 218 221 15654036 10.1136/jnnp.2004.041202 PMC1739520

[B15] Schoenen J Jensen RH Lantéri-Minet M Láinez MJ Gaul C Goodman AM Stimulation of the sphenopalatine ganglion (SPG) for cluster headache treatment. Pathway CH-1: A randomized, sham-controlled study Cephalalgia 2013 doi: 10.1177/0333102412473667 10.1177/0333102412473667 PMC3724276 23314784

[B16] Pedersen JL Barloese M Jensen RH Neurostimulation in cluster headache: A review of current progress Cephalalgia 2013 [Epub ahead of print] doi: 10.1177/0333102413489040 10.1177/0333102413489040 23687279

[B17] Leone M May A Franzini A Broggi G Dodick D Rapoport A Goadsby PJ Schoenen J Bonavita V Bussone G Deep brain stimulation for intractable chronic cluster headache: proposals for patient selection Cephalalgia 2004 24 934 7 15482354 10.1111/j.1468-2982.2004.00742.x

[B18] May A Bahra A Büchel C Frackowiak RSJ Goadsby PJ Hypothalamic activation in cluster headache attacks Lancet 1998 352 275 78 9690407 10.1016/S0140-6736(98)02470-2

[B19] May A Ashburner J Buchel C Correlation between structural and functional changes in brain in an idiopathic headache syndrome Nat Med 1999 5 836 38 10395332 10.1038/10561

[B20] Leone M Franzini A Bussone G Stereotactic stimulation of posterior hypothalamic gray matter in a patient with intractable cluster headache N Engl J Med 2001 345 1428 29 11794190 10.1056/NEJM200111083451915

[B21] Schoenen J Di Clemente L Vandenheede M Hypothalamic stimulation in chronic cluster headache: a pilot study of efficacy and mode of action Brain 2005 128 940 47 15689358 10.1093/brain/awh411

[B22] Leone M Franzini A Broggi G Bussone G Hypothalamic stimulation for intractable cluster headache: long-term experience Neurology 2006 67 150 52 16832097 10.1212/01.wnl.0000223319.56699.8a

[B23] D’Andrea G Nordera G Piacentino M Effectiveness of hypothalamic stimulation in two patients affected by intractable chronic cluster headache Neurology 2006 66 suppl 2 A140 abstr

[B24] Benabid A Seigneuret E Torres N Intraventricular stimulation for targets close to the midline: periaqueductal gray, posterior hypothalamus, anterior hypothalamus, subcommissural structures Acta Neurochir 2006 148 1 64 abstr16328771

[B25] Starr PA Barbaro NM Raskin NH Ostrem JL Chronic stimulation of the posterior hypothalamic region for cluster headache: technique and 1-year results in four patients J Neurosurg 2007 106 999 1005 17564171 10.3171/jns.2007.106.6.999

[B26] Owen SL Green AL Davies P Connectivity of an effective hypothalamic surgical target for cluster headache J Clin Neurosci 2007 14 10 955 60 17689083 10.1016/j.jocn.2006.07.012

[B27] Black D Bartleson J Torgrimson S Davis D Two cases of chronic cluster headache treated successfully with hypothalamic deep brain stimulation Neurology (abstract) 2007 68 1 A307

[B28] Mateos V Seijo F Lozano B Deep brain stimulation in chronic refractory headaches: first national cases Neurologia (abstract) 2007 22 96

[B29] Bartsch T Pinsker MO Rasche D Hypothalamic deep brain stimulation for cluster headache: experience from a new multicase series Cephalalgia 2008 28 285 95 18254897 10.1111/j.1468-2982.2007.01531.x

[B30] Fontaine D Lazorthes Y Mertens P Safety and efficacy of deep brain stimulation in refractory cluster headache: a randomized placebo-controlled double-blind trial followed by a 1-year open extension J Headache Pain 2010 11 23 31 19936616 10.1007/s10194-009-0169-4 PMC3452182

[B31] Hidding U May A Mere surgery will not cure cluster headache: implications for neurostimulation Cephalalgia 2011 31 112 15 20974592 10.1177/0333102410373157

[B32] Seijo F Saiz A Lozano B Neuromodulation of the posterolateral hypothalamus for the treatment of chronic refractory cluster headache: experience in five patients with a modified anatomical target Cephalalgia 2011 31 1634 41 22116943 10.1177/0333102411430264

[B33] Leone M Franzini A Proietti Cecchini A Bussone G Success, failure, and putative mechanisms in hypothalamic stimulation for drug-resistant chronic cluster headache Pain 2013 154 89 94 23103434 10.1016/j.pain.2012.09.011

[B34] Leone M Franzini A D’Andrea G Broggi G Casucci G Bussone G Deep brain stimulation to relieve drug-resistant SUNCT Ann Neurol 2005 57 924 27 15929061 10.1002/ana.20507

[B35] Lyons MK Dodick DW Evidente VG Responsiveness of short-lasting unilateral neuralgiform headache with conjunctival injection and tearing to hypothalamic deep brain stimulation J Neurosurg 2009 110 279 81 18821829 10.3171/2008.4.17493

[B36] Bartsch T Falk D Knudsen K Deep brain stimulation of the posterior hypothalamic area in intractable short-lasting unilateral neuralgiform headache with conjunctival injection and tearing (SUNCT) Cephalalgia 2011 31 1405 08 21628443 10.1177/0333102411409070

[B37] Walcott BP Bamber NI Anderson DE Successful treatment of chronic paroxysmal hemicrania with posterior hypothalamic stimulation: technical case report Neurosurgery 2009 65 E997 19834392 10.1227/01.NEU.0000345937.05186.73

[B38] Headache classification subcommittee of the International Headache Society The International Classification of Headache Disorders, 2nd edn Cephalalgia 2004 24 suppl 1 9 160 14979299 10.1111/j.1468-2982.2003.00824.x

[B39] Leone M Franzini A Broggi G Mea E Cecchini AP Bussone G Acute hypothalamic stimulation and ongoing cluster headache attacks Neurology 2006 67 1844 45 17130420 10.1212/01.wnl.0000247273.93084.49

[B40] The deep-brain stimulation for Parkinson’s disease group Deep-brain stimulation on the subthalamic nucleus or pars interna of the globus pallidus in Parkinson’s disease N Engl J Med 2001 345 956 96335 11575287 10.1056/NEJMoa000827

[B41] Fontaine D Lanteri-Minet M Ouchchane L Anatomical location of effective deep brain stimulation electrodes in chronic cluster headache Brain 2010 133 1214 23 20237130 10.1093/brain/awq041

[B42] Maniyar FH Starr P Goadsby PJ Paroxysmal sneezing after hypothalamic deep brain stimulation for cluster headache Cephalalgia 2012 32 8 641 644 22529193 10.1177/0333102412442412

[B43] Cortelli P Guaraldi P Leone M Effect of deep brain stimulation of the posterior hypothalamic area on the cardiovascular system in chronic cluster headache patients Eur J Neurol 2007 14 1008 1536 17718693 10.1111/j.1468-1331.2007.01850.x

[B44] Vetrugno R Pierangeli G Leone M Effect on sleep of posterior hypothalamus stimulation in cluster headache Headache 2007 47 1085 90 17635601 10.1111/j.1526-4610.2007.00864.x

[B45] Jürgens T Leone M Proietti-Cecchini A Hypothalamic deep-brain stimulation modulates thermal sensitivity and pain thresholds in cluster headache Pain 2009 146 84 90 19679396 10.1016/j.pain.2009.07.006

[B46] May A Leone M Boecker H Hypothalamic deep brain stimulation in positron emission tomography J Neurosci 2006 26 3589 93 16571767 10.1523/JNEUROSCI.4609-05.2006 PMC6673857

[B47] May A Chronic pain may change the structure of the brain Pain 2008 137 7 15 18410991 10.1016/j.pain.2008.02.034

[B48] Zhuo M Cortical excitation and chronic pain Trends Neurosci 2008 31 199 207 18329111 10.1016/j.tins.2008.01.003

[B49] Bartsch T Goadsby PJ Stimulation of the greater occipital nerve induces increased central excitability of dural afferent input Brain 2002 125 1496 509 12077000 10.1093/brain/awf166

[B50] Bartsch T Goadsby PJ Increased responses in trigeminocervical nociceptive neurons to cervical input after stimulation of the dura mater Brain 2003 126 1801 13 12821523 10.1093/brain/awg190

[B51] Anthony M Headache and the greater occipital nerve Clinical neurology and neurosurgery 1992 94 297 301 1335856 10.1016/0303-8467(92)90177-5

[B52] Ambrosini A Vandenheede M Rossi P Aloj F Sauli E Pierelli F Suboccipital injection with a mixture of rapid- and long-acting steroids in cluster headache: a double-blind placebo-controlled study Pain 2005 118 92 6 16202532 10.1016/j.pain.2005.07.015

[B53] Afridi SK Shields KG Bhola R Goadsby PJ Greater occipital nerve injection in primary headache syndromes–prolonged effects from a single injection Pain 2006 122 126 9 16527404 10.1016/j.pain.2006.01.016

[B54] Weiner RL Reed KL Peripheral neurostimulation for control of intractable occipital neuralgia Neuromodulation 1999 2 217 22 22151211 10.1046/j.1525-1403.1999.00217.x

[B55] Lipton R Goadsby P Cady R Aurora SK Grosberg B Freitag F PRISM study: occipital nerve stimulation for treatment-refractory migraine Cephalalgia 2009 29 30

[B56] Saper JR Dodick DW Silberstein SD McCarville S Sun M Goadsby PJ Occipital nerve stimulation for the treatment of intractable chronic migraine headache: ONSTIM feasibility study Cephalalgia 2011 31 271 85 20861241 10.1177/0333102410381142 PMC3057439

[B57] Silberstein S Dodick D Saper J Huh B Slavin KV Sharan A Safety and efficacy of peripheral nerve stimulation of the occipital nerves for the management of chronic migraine: Results from a randomized, multicenter, double-blinded, controlled study Cephalalgia 2012 32 16 1165 79 23034698 10.1177/0333102412462642

[B58] Magis D Schoenen J Advances and challenges in neurostimulation for headaches Lancet Neurol 2012 11 708 19 22814542 10.1016/S1474-4422(12)70139-4

[B59] Reed KL Black SB Banta CJ 2nd Will KR Combined occipital and supraorbital neurostimulation for the treatment of chronic migraine headaches: initial experience Cephalalgia 2010 30 260 71 19732075 10.1111/j.1468-2982.2009.01996.x

[B60] Burns B Watkins L Goadsby PJ Treatment of intractable chronic cluster headache by occipital nerve stimulation in 14 patients Neurology 2009 72 341 5 19171831 10.1212/01.wnl.0000341279.17344.c9

[B61] Magis D Gerardy PY Remacle JM Schoenen J Sustained effectiveness of occipital nerve stimulation in drug-resistant chronic cluster headache Headache 2011 51 1191 201 21848953 10.1111/j.1526-4610.2011.01973.x

[B62] Magis D Allena M Bolla M De Pasqua V Remacle JM Schoenen J Occipital nerve stimulation for drug-resistant chronic cluster headache: a prospective pilot study Lancet Neurol 2007 6 314 21 17362835 10.1016/S1474-4422(07)70058-3

[B63] Fontaine D Christophe Sol J Raoul S Fabre N Geraud G Magne C Treatment of refractory chronic cluster headache by chronic occipital nerve stimulation Cephalalgia 2011 31 1101 5 21727143 10.1177/0333102411412086

[B64] Burns B Watkins L Goadsby PJ Treatment of hemicrania continua by occipital nerve stimulation with a bion device: long-term follow-up of a crossover study Lancet Neurol 2008 7 1001 12 18845482 10.1016/S1474-4422(08)70217-5

[B65] Marin JC Goadsby P Response of SUNCT (Short-Lasting Unilateral Neuralgiform Headaches with Conjunctival Injection and Tearing), SUNA (Short-LAsting Unilateral Neuralgifom Headaches with Autonomic Symptoms) and Primary Stabbing Headaches to Occipital Nerve Stimulation (ONS) Neurology 2010 74 P04.006 abstract

[B66] Shanahan P Watkins L Matharu M Treatment of medically intractable short- lasting unilateral neuralgiform headache attacks with conjunctival injection and tearing (SUNCT) and short-lasting unilateral neuralgiform headache attacks with autonomic symptoms (SUNA) with occipital nerve stimulation (ONS) in 6 patients Cephalalgia 2009 29 150

[B67] Magis D Bruno MA Fumal A Gerardy PY Hustinx R Laureys S Central modulation in cluster headache patients treated with occipital nerve stimulation: an FDG-PET study BMC Neurol 2011 11 25 21349186 10.1186/1471-2377-11-25 PMC3056751

[B68] Matharu MS Bartsch T Ward N Frackowiak RS Weiner R Goadsby PJ Central neuromodulation in chronic migraine patients with suboccipital stimulators: a PET study Brain 2004 127 220 30 14607792 10.1093/brain/awh022

[B69] Natsis K Baraliakos X Appell HJ Tsikaras P Gigis I Koebke J The course of the greater occipital nerve in the suboccipital region: a proposal for setting landmarks for local anesthesia in patients with occipital neuralgia Clinical anatomy 2006 19 332 6 16258972 10.1002/ca.20190

[B70] Paemeleire K Van Buyten JP Van Buynder M Alicino D Van Maele G Smet I Phenotype of patients responsive to occipital nerve stimulation for refractory head pain Cephalalgia 2010 30 662 73 20511204 10.1111/j.1468-2982.2009.02022.x

[B71] May A Headaches with (ipsilateral) autonomic symptoms J Neurol 2003 250 1273 8 14648142 10.1007/s00415-003-0241-y

[B72] Devoghel JC Cluster headache and sphenopalatine block Acta Anaesthesiol Belg 1981 32 101 7 7293708

[B73] Felisati G Arnone F Lozza P Leone M Curone M Bussone G Sphenopalatine endoscopic ganglion block: a revision of a traditional technique for cluster headache Laryngoscope 2006 116 1447 50 16885751 10.1097/01.mlg.0000227997.48020.44

[B74] Narouze S Kapural L Casanova J Mekhail N Sphenopalatine ganglion radiofrequency ablation for the management of chronic cluster headache Headache 2009 49 571 7 18783451 10.1111/j.1526-4610.2008.01226.x

[B75] Ruskell GL Orbital passage of pterygopalatine ganglion efferents to paranasal sinuses and nasal mucosa in man Cells Tissues Organs 2003 175 223 28 14707402 10.1159/000074943

[B76] Oomen KP Van Wijck AJ Hordijk GJ De Ru JA Effects of radiofrequency thermocoagulation of the sphenopalatine ganglion on headache and facial pain: correlation with diagnosis J Orofac Pain 2012 26 59 64 22292141

[B77] Narouze SN Role of sphenopalatine ganglion neuroablation in the management of cluster headache Curr Pain Headache Rep 2010 14 160 3 20425206 10.1007/s11916-010-0100-3

[B78] Sanders M Zuurmond WW Efficacy of sphenopalatine ganglion blockade in 66 patients suffering from cluster headache: a 12- to 70-month follow-up evaluation J Neurosurg 1997 87 876 80 9384398 10.3171/jns.1997.87.6.0876

[B79] Blumenfeld A Ashkenazi A Napchan U Bender SD Klein BC Berliner R Expert Consensus Recommendations for the Performance of Peripheral Nerve Blocks for Headaches - A Narrative Review Headache 2013 53 437 46 23406160 10.1111/head.12053

[B80] Ansarinia M Rezai A Tepper SJ Steiner CP Stump J Stanton-Hicks M Electrical stimulation of sphenopalatine ganglion for acute treatment of cluster headaches Headache 2010 50 1164 74 20438584 10.1111/j.1526-4610.2010.01661.x

[B81] Tepper SJ Rezai A Narouze S Steiner C Mohajer P Ansarinia M Acute treatment of intractable migraine with sphenopalatine ganglion electrical stimulation Headache 2009 49 983 9 19486173 10.1111/j.1526-4610.2009.01451.x

[B82] Schytz HW Barløse M Guo S Selb J Caparso A Jensen R Experimental activation of the sphenopalatine ganglion provokes cluster-like attacks in humans Cephalalgia 2013 10.1177/0333102413476370 23382519

[B83] Ammons WS Blair RW Foreman RD Vagal afferent inhibition of primate thoracic spinothalamic neurons J Neurophysiol 1983 50 926 940 6631470 10.1152/jn.1983.50.4.926

[B84] Thies R Foreman RD Inhibition and excitation of thoracic spinoreticular neurons by electrical stimulation of vagal afferent nerves Exp Neurol 1983 82 1 16 6628602 10.1016/0014-4886(83)90238-8

[B85] Maixner W Randich A Role of the right vagal nerve trunk in antinociception Brain Res 1984 298 374 377 6326956 10.1016/0006-8993(84)91441-0

[B86] Ren K Randich A Gebhart GF Vagal afferent modulation of spinal nociceptive transmission in the rat J Neurophysiol 1989 62 401 415 2549208 10.1152/jn.1989.62.2.401

[B87] Randich A Ren K Gebhart GF Electrical stimulation of cervical vagal afferents. II. Central relays for behavioral antinociception and arterial blood pressure decreases J Neurophysiol 1990 64 1115 1124 2258737 10.1152/jn.1990.64.4.1115

[B88] Chandler MJ Hobbs SF Bolser DC Foreman RD Effects of vagal afferent stimulation on cervical spinothalamic tract neurons in monkeys Pain 1991 44 81 87 2038494 10.1016/0304-3959(91)90152-N

[B89] Aicher SA Lewis SJ Randich A Antinociception produced by electrical stimulation of vagal afferents: independence of cervical and subdiaphragmatic branches Brain Res 1991 542 63 70 2054659 10.1016/0006-8993(91)90998-b

[B90] Thurston CL Randich A Quantitative characterization and spinal substrates of antinociception produced by electrical stimulation of the subdiaphragmatic vagus in rats Pain 1991 44 201 209 2052387 10.1016/0304-3959(91)90138-N

[B91] Thurston CL Randich A Effects of vagal afferent stimulation on ON and OFF cells in the rostroventral medulla: Relationships to nociception and arterial blood pressure J Neurophysiol 1992 67 180 196 1552318 10.1152/jn.1992.67.1.180

[B92] Rutecki P Anatomical, physiological, and theoretical basis for the antiepileptic effect of vagus nerve stimulation Epilepsia 1990 31 Suppl 2 S1 6 2226360 10.1111/j.1528-1157.1990.tb05843.x

[B93] Bossut DF Maixner W Effects of cardiac vagal afferent electrostimulation on the response of trigeminal and trigeminothalamic neurons to noxious orofacial stimulation Pain 1996 65 101 109 8826496 10.1016/0304-3959(95)00166-2

[B94] Evans AR Jones SL Blair RW Effects of vagal afferent nerve stimulation on noxious heat-evoked Fos-like immunoreactivity in the rat lumbar spinal cord J Comp Neurol 1994 22 490 498 7983241 10.1002/cne.903460403

[B95] Nishikawa Y Koyama N Yoshida Y Yokota T Activation of ascending antinociceptive system by vagal afferent input as revealed in the nucleus ventralis posteromedialis Brain Res 1999 833 108 111 10375683 10.1016/s0006-8993(99)01521-8

[B96] Hord ED Evans MS Mueed S Adamolekun B Naritoku DK The effect of vagus nerve stimulation on migraines J Pain 2003 4 530 534 14636821 10.1016/j.jpain.2003.08.001

[B97] Lenaerts ME Oommen KJ Couch JR Skaggs V Can vagus nerve stimulation help migraine? Cephalalgia 2008 28 392 395 18279429 10.1111/j.1468-2982.2008.01538.x

[B98] Mauskop A Vagus nerve stimulation relieves chronic refractory migraine and cluster headaches Cephalalgia 2005 25 82 86 15658944 10.1111/j.1468-2982.2005.00611.x

[B99] Ventureyra EC Transcutaneous vagus nerve stimulation for partial onset seizure therapy. A new concept Childs Nerv Syst 2000 16 101 102 10663816 10.1007/s003810050021

[B100] Fallgatter AJ Neuhauser B Herrmann MJ Ehlis AC Wagener A Scheuerpflug P Reiners K Riederer P Far field potentials from the brain stem after transcutaneous vagus nerve stimulation J Neural Transm 2003 110 12 1437 1443 14666414 10.1007/s00702-003-0087-6

[B101] Fallgatter AJ Ehlis AC Ringel TM Herrmann MJ Age effect on far field potentials from the brain stem after transcutaneous vagus nerve stimulation Int J Psychophysiol 2005 56 1 37 43 15725488 10.1016/j.ijpsycho.2004.09.007

[B102] Nesbitt AD Marin JCA Tomkins E Ruttledge MH Goadsby PJ Non-invasive vagus nerve stimulation for the treatment of cluster headache: a case series J Headache Pain 2013 1 Suppl 1 P231

[B103] Magis D Gérard P Schoenen J Transcutaneous Vagus Nerve Stimulation (tVNS) for headache prophylaxis: initial experience J Headache Pain 2013 1 Suppl 1 P198

[B104] Nitsche MA Paulus W Transcranial direct current stimulation update 2011 Restor Neurol Neurosci 2011 29 463 492 22085959 10.3233/RNN-2011-0618

[B105] Antal A Paulus W Nitsche MA Electrical stimulation and visual network plasticity Restor Neurol Neurosci 2011 29 365 374 22124032 10.3233/RNN-2011-0609

[B106] Bindman LJ Lippold OC Redfearn JW The Action of Brief Polarizing Currents on the Cerebral Cortex of the Rat (1) during Current Flow and (2) in the Production of Long-Lasting after-Effects J Physiol 1964 172 369 382 14199369 10.1113/jphysiol.1964.sp007425 PMC1368854

[B107] Bohotin V Fumal A Vandenheede M Gerard P Bohotin C Maertens de Noordhout A Schoenen J Effects of repetitive transcranial magnetic stimulation on visual evoked potentials in migraine Brain 2002 125 912 922 11912123 10.1093/brain/awf081

[B108] Brunoni AR Amadera J Berbel B Volz MS Rizzerio BG Fregni F A systematic review on reporting and assessment of adverse effects associated with transcranial direct current stimulation Int J Neuropsychopharmacol 2011 14 1133 1145 21320389 10.1017/S1461145710001690

[B109] Creutzfeldt OD Fromm GH Kapp H Influence of transcortical d-c currents on cortical neuronal activity Exp Neurol 1962 5 436 452 13882165 10.1016/0014-4886(62)90056-0

[B110] Nitsche MA Paulus W Excitability changes induced in the human motor cortex by weak transcranial direct current stimulation J Physiol 2000 527 Pt 3 633 639 10990547 10.1111/j.1469-7793.2000.t01-1-00633.x PMC2270099

[B111] Nitsche MA Paulus W Sustained excitability elevations induced by transcranial DC motor cortex stimulation in humans Neurology 2001 57 1899 1901 11723286 10.1212/wnl.57.10.1899

[B112] Liebetanz D Nitsche MA Tergau F Paulus W Pharmacological approach to the mechanisms of transcranial DC-stimulation-induced after-effects of human motor cortex excitability Brain 2002 125 2238 2247 12244081 10.1093/brain/awf238

[B113] Antal A Paulus W A case of refractory orofacial pain treated by transcranial direct current stimulation applied over hand motor area in combination with NMDA agonist drug intake Brain Stimul 2011 4 117 121 21511214 10.1016/j.brs.2010.09.003

[B114] Antal A Kriener N Lang N Boros K Paulus W Cathodal transcranial direct current stimulation of the visual cortex in the prophylactic treatment of migraine Cephalalgia 2011 31 820 828 21398419 10.1177/0333102411399349

[B115] Auvichayapat P Janyacharoen T Rotenberg A Tiamkao S Krisanaprakornkit T Sinawat S Punjaruk W Thinkhamrop B Auvichayapat N Migraine prophylaxis by anodal transcranial direct current stimulation, a randomized, placebo-controlled trial J Med Assoc Thai 2012 95 1003 1012 23061303

[B116] Dasilva AF Mendonca ME Zaghi S Lopes M Dossantos MF Spierings EL Bajwa Z Datta A Bikson M Fregni F tDCS-induced analgesia and electrical fields in pain-related neural networks in chronic migraine Headache 2012 52 1283 1295 22512348 10.1111/j.1526-4610.2012.02141.x PMC4166674

[B117] Priori A Hallett M Rothwell JC Repetitive transcranial magnetic stimulation or transcranial direct current stimulation? Brain Stimul 2009 2 241 245 20633424 10.1016/j.brs.2009.02.004

[B118] Poreisz C Boros K Antal A Paulus W Safety aspects of transcranial direct current stimulation concerning healthy subjects and patients Brain Res Bull 2007 72 208 214 17452283 10.1016/j.brainresbull.2007.01.004

[B119] Ambrus GG Al-Moyed H Chaieb L Sarp L Antal A Paulus W The fade-in–short stimulation–fade out approach to sham tDCS–reliable at 1 mA for naive and experienced subjects, but not investigators Brain Stimul 2012 5 499 504 22405745 10.1016/j.brs.2011.12.001

[B120] Nitsche MA Fricke K Henschke U Schlitterlau A Liebetanz D Lang N Henning S Tergau F Paulus W Pharmacological modulation of cortical excitability shifts induced by transcranial direct current stimulation in humans J Physiol 2003 553 293 301 12949224 10.1113/jphysiol.2003.049916 PMC2343495

[B121] Nitsche MA Grundey J Liebetanz D Lang N Tergau F Paulus W Catecholaminergic consolidation of motor cortical neuroplasticity in humans Cereb Cortex 2004 14 1240 1245 15142961 10.1093/cercor/bhh085

[B122] Nitsche MA Liebetanz D Lang N Antal A Tergau F Paulus W Safety criteria for transcranial direct current stimulation (tDCS) in humans Clinical neurophysiology: official journal of the International Federation of Clinical Neurophysiology 2003 114 2220 2222 author reply 2222–2223 14580622 10.1016/s1388-2457(03)00235-9

[B123] Barker AT Jalinous R Freeston IL Non-invasive magnetic stimulation of human motor cortex Lancet 1985 1 1106 7 2860322 10.1016/s0140-6736(85)92413-4

[B124] Thickbroom GW Transcranial magnetic stimulation and synaptic plasticity: experimental framework and human models Exp Brain Res 2007 180 583 93 17562028 10.1007/s00221-007-0991-3

[B125] Martens JW Koehler PJ Vijselaar J Magnetic flimmers: 'light in the electromagnetic darkness' Brain 2013 136 971 9 23043145 10.1093/brain/aws185

[B126] Coppola G Pierelli F Schoenen J Is the cerebral cortex hyperexcitable or hyperresponsive in migraine? Cephalalgia 2007 27 1427 39 18034686 10.1111/j.1468-2982.2007.01500.x

[B127] Siniatchkin M Sendacki M Moeller F Abnormal changes of synaptic excitability in migraine with aura Cereb Cortex 2012 22 2207 16 22079926 10.1093/cercor/bhr248

[B128] Brighina F Palermo A Fierro B Cortical inhibition and habituation to evoked potentials: relevance for pathophysiology of migraine J Headache Pain 2009 10 77 84 19209386 10.1007/s10194-008-0095-x PMC3451650

[B129] Bernstein C Burstein R Sensitization of the trigeminovascular pathway: perspective and implications to migraine pathophysiology J Clin Neurol 2012 8 89 99 22787491 10.3988/jcn.2012.8.2.89 PMC3391624

[B130] Moulton EA Burstein R Tully S Hargreaves R Becerra L Borsook D Interictal dysfunction of a brainstem descending modulatory center in migraine patients PLoS One 2008 3 e3799 19030105 10.1371/journal.pone.0003799 PMC2582961

[B131] Lipton RB Dodick DW Silberstein SD Saper JR Aurora SK Pearlman SH Fischell RE Ruppel PL Goadsby PJ Single-pulse transcranial magnetic stimulation for acute treatment of migraine with aura: a randomised, double-blind, parallel-group, sham-controlled trial Lancet Neurol 2010 9 373 80 20206581 10.1016/S1474-4422(10)70054-5

[B132] Brighina F Piazza A Vitello G rTMS of the prefrontal cortex in the treatment of chronic migraine: a pilot study J Neurol Sci 2004 227 67 71 15546593 10.1016/j.jns.2004.08.008

[B133] Lorenz J Minoshima S Casey KL Keeping pain out of mind: the role of the dorsolateral prefrontal cortex in pain modulation Brain 2003 126 1079 91 12690048 10.1093/brain/awg102

[B134] Misra UK Kalita J Tripathi GM Bhoi SK Is β endorphin related to migraine headache and its relief? Cephalalgia 2013 33 5 316 22 23314782 10.1177/0333102412473372

[B135] Zaghi S Thiele B Pimentel D Assessment and treatment of pain with non-invasive cortical stimulation Restor Neurol Neurosci 2011 29 439 51 22124038 10.3233/RNN-2011-0615

[B136] Dodick DW Schembri CT Helmuth M Transcranial magnetic stimulation for migraine: a safety review Headache 2010 50 1153 63 20553334 10.1111/j.1526-4610.2010.01697.x

[B137] Richards EM Payne JL The management of mood disorders in pregnancy: alternatives to antidepressants CNS Spectr 2013 Apr 10 1 11 10.1017/S1092852913000151 23570692

[B138] Brighina F Palermo A Daniele O High-frequency transcranial magnetic stimulation on motor cortex of patients affected by migraine with aura: a way to restore normal cortical excitability? Cephalalgia 2010 30 46 52 19438928 10.1111/j.1468-2982.2009.01870.x

[B139] Brighina F Cosentino G Vigneri S Talamanca S Palermo A Giglia G Fierro B Abnormal facilitatory mechanisms in motor cortex of migraine with aura Eur J Pain 2011 15 928 35 21530338 10.1016/j.ejpain.2011.03.012

[B140] de Tommaso M Stramaglia S Brighina F Fierro B Francesco VD Todarello O Serpino C Pellicoro M Lack of effects of low frequency repetitive transcranial magnetic stimulation on alpha rhythm phase synchronization in migraine patients Neurosci Lett 2011 20 143 7 21075171 10.1016/j.neulet.2010.11.017

[B141] Fumal A Coppola G Bohotin V Induction of long-lasting changes of visual cortex excitability by five daily sessions of repetitive transcranial magnetic stimulation (rTMS) in healthy volunteers and migraine patients Cephalalgia 2006 26 143 149 16426268 10.1111/j.1468-2982.2005.01013.x

[B142] de Tommaso M Brighina F Fierro B Effects of high-frequency repetitive transcranial magnetic stimulation of primary motor cortex on laser-evoked potentials in migraine J Headache Pain 2010 11 505 12 20714776 10.1007/s10194-010-0247-7 PMC3476225

[B143] Nnoaham KE Kumbang J Transcutaneous electrical nerve stimulation (TENS) for chronic pain Cochrane Database Syst Rev 2008 16 3 CD003222 18646088 10.1002/14651858.CD003222.pub2

[B144] Han JS Chen XH Sun SL Xu XJ Yuan Y Yan SC Hao JX Terenius L Effect of low- and high-frequency TENS on Met-enkephalin-Arg-Phe and dynorphin A immunoreactivity in human lumbar CSF Pain 1991 47 295 8 1686080 10.1016/0304-3959(91)90218-M

[B145] Walsh DM Howe TE Johnson MI Sluka KA Transcutaneous electrical nerve stimulation for acute pain Cochrane Database Syst Rev 2009 15 2 CD006142 19370629 10.1002/14651858.CD006142.pub2

[B146] Hurlow A Bennett MI Robb KA Johnson MI Simpson KH Oxberry SG Transcutaneous electric nerve stimulation (TENS) for cancer pain in adults Cochrane Database Syst Rev 2012 3 CD006276 22419313 10.1002/14651858.CD006276.pub3 PMC6669272

[B147] Khadilkar A Odebiyi DO Brosseau L Wells GA Transcutaneous electrical nerve stimulation (TENS) versus placebo for chronic low-back pain Cochrane Database Syst Rev 2008 8 4 CD003008 18843638 10.1002/14651858.CD003008.pub3 PMC7138213

[B148] Dubinsky RM Miyasaki J Assessment: efficacy of transcutaneous electric nerve stimulation in the treatment of pain in neurologic disorders (an evidence-based review): report of the Therapeutics and Technology Assessment Subcommittee of the American Academy of Neurology Neurology 2010 74 173 6 20042705 10.1212/WNL.0b013e3181c918fc

[B149] Appenzeller O Atkinson R Transcutaneous nerve stimulation for the treatment of migraine and other head pain (author’s transl) MMW Munch Med Wochenschr 1975 117 1953 4 813129

[B150] Solomon S Guglielmo KM Treatment of headache by transcutaneous electrical stimulation Headache 1985 25 12 5 3871754 10.1111/j.1526-4610.1985.hed2501012.x

[B151] Bronfort G Nilsson N Haas M Evans R Goldsmith CH Assendelft WJ Bouter LM Non-invasive physical treatments for chronic/recurrent headache Cochrane Database Syst Rev 2004 1 3 CD001878 15266458 10.1002/14651858.CD001878.pub2

[B152] Mousavi SA Mirbod SM Khorvash F Comparison between efficacy of imipramine and transcutaneous electrical nerve stimulation in the prophylaxis of chronic tension-type headache: a randomized controlled clinical trial J Res Med Sci 2011 16 923 27 22279461 PMC3263106

[B153] Schoenen J Vandersmissen B Jeangette S Herroelen L Vandenheede M Gerard P Magis D Prevention of migraine by supraorbital transcutaneous neurostimulation using the Cefaly device (PREMICE): a multi-centre, randomized, sham-controlled trial J Headache Pain 2013 1 Suppl 1 P184

[B154] Ellens DJ Levy RM Peripheral neuromodulation for migraine headache Progress in neurological surgery 2011 24 109 117 doi: 10.1159/000323890 21422781 10.1159/000323890

[B155] Goadsby PJ Charbit AR Andreou AP Akerman S Holland PR Neurobiology of migraine Neuroscience 2009 161 327 341 doi: 10.1016/j.neuroscience.2009.03.019 19303917 10.1016/j.neuroscience.2009.03.019

[B156] Tomycz ND Deibert CP Moossy JJ Cervicomedullary Junction Spinal Cord Stimulation for Head and Facial Pain Headache: The Journal of Head and Face Pain 2011 51 418 425 doi: 10.1111/j.1526-4610.2010.01829.x 10.1111/j.1526-4610.2010.01829.x 21269299

[B157] Wolter T Kiemen A Kaube H High cervical spinal cord stimulation for chronic cluster headache Cephalalgia 2011 31 11 1170 1180 doi: 10.1177 /0333102411412627 21700642 10.1177/0333102411412627

[B158] Gaul C Jurgens T May A Concerning high cervical spinal cord stimulation for chronic cluster headache Cephalalgia 2011 31 15 1588 1589 doi: 10.1177/0333102411422384 21914733 10.1177/0333102411422384

[B159] Palmisani S Al-Kaisy A Arcioni R Smith T Negro A Lambru G Bandikatla V Carson E Martelletti P A six year retrospective review of occipital nerve stimulation practice - controversies and challenges of an emerging technique for treating refractory headache syndromes J Headache Pain 2013 14 1 67 doi: 10.1186/1129-2377-14-67 23919570 10.1186/1129-2377-14-67 PMC3751236

[B160] Goadsby P Analysis of occipital nerve stimulation in studies of chronic migraine and broader implications of social media in clinical trials Cephalalgia 2012 33 214 215 23212293 10.1177/0333102412468680

